# Cocaine Is Low on the Value Ladder of Rats: Possible Evidence for Resilience to Addiction

**DOI:** 10.1371/journal.pone.0011592

**Published:** 2010-07-28

**Authors:** Lauriane Cantin, Magalie Lenoir, Eric Augier, Nathalie Vanhille, Sarah Dubreucq, Fuschia Serre, Caroline Vouillac, Serge H. Ahmed

**Affiliations:** Unité Mixte de Recherche 5227, Centre National de la Recherche Scientifique, Université Victor Segalen Bordeaux 2, Bordeaux, France; Chiba University Center for Forensic Mental Health, Japan

## Abstract

**Background:**

Assessing the relative value of cocaine and how it changes with chronic drug use represents a long-standing goal in addiction research. Surprisingly, recent experiments in rats – by far the most frequently used animal model in this field – suggest that the value of cocaine is lower than previously thought.

**Methodology/Principal Findings:**

Here we report a series of choice experiments that better define the relative position of cocaine on the value ladder of rats (i.e., preference rank-ordering of different rewards). Rats were allowed to choose either taking cocaine or drinking water sweetened with saccharin – a nondrug alternative that is not biologically essential. By systematically varying the cost and concentration of sweet water, we found that cocaine is low on the value ladder of the large majority of rats, near the lowest concentrations of sweet water. In addition, a retrospective analysis of all experiments over the past 5 years revealed that no matter how heavy was past cocaine use most rats readily give up cocaine use in favor of the nondrug alternative. Only a minority, fewer than 15% at the heaviest level of past cocaine use, continued to take cocaine, even when hungry and offered a natural sugar that could relieve their need of calories.

**Conclusions/Significance:**

This pattern of results (cocaine abstinence in most rats; cocaine preference in few rats) maps well onto the epidemiology of human cocaine addiction and suggests that only a minority of rats would be vulnerable to cocaine addiction while the large majority would be resilient despite extensive drug use. Resilience to drug addiction has long been suspected in humans but could not be firmly established, mostly because it is difficult to control retrospectively for differences in drug self-exposure and/or availability in human drug users. This conclusion has important implications for preclinical research on the neurobiology of cocaine addiction and for future medication development.

## Introduction

The immediate reward value of cocaine, especially if it is rapidly delivered to the brain following smoking or intravenous injection, is widely thought to be higher than that of most natural or socially-valued rewards – a difference that would contribute to explain its addictive potential [1]–[5]. This assumption is largely based on retrospective self-reports from current or ex-cocaine addicts or on evidence from experimental animals given access to cocaine self-administration with no behavioral alternative available. It seems also to be corroborated, though more indirectly, by neurobiological research showing that cocaine provokes a surge of dopamine in the ventral striatum that is abnormally high and that does not habituate to repeated drug exposure, compared to that evoked by nondrug rewards [3], [5], [6]. However, estimating the relative value of cocaine in current or ex-cocaine abusers – who belong to a non-representative minority – is prone to a selection bias and is thus likely to lead to overestimates when generalized to the majority of other, unselected populations. There is no doubt that cocaine can be initially highly rewarding in some vulnerable individuals [7]–[10]; whether this is true in the large majority of other unselected individuals remains to be demonstrated [11]–[13]. Similarly, though there is no doubt that most experimental animals readily self-administer cocaine when no other valuable choices are available, this evidence in itself does not provide information about its relative value compared to that of other nondrug rewards. As a matter of fact, since the seminal work by Pickens and Thompson in 1968 [14], comparatively little research has been conducted in experimental animals to quantity the relative value of cocaine (i.e., in comparison to nondrug reward) [15], [16].

Recent research in (unselected) rats – by far the most frequently used animal model in experimental addiction research [17] – has revealed that the relative value of cocaine is surprisingly weaker than previously thought [18]–[21]. For instance, using a reliable behavioral economic approach, it was recently estimated in hungry rats from different strains that the reward value of food is largely greater than the reward value of intravenous cocaine [18], [19], a difference that persisted even following long-term cocaine self-administration [20]. Considering that food is essential for survival, growth and reproduction, this outcome may not be surprising. Perhaps more surprisingly, we found that when offered a mutually-exclusive choice, most non-deprived rats readily give up cocaine use to drink water sweetened with a non-caloric sweetener (i.e., saccharin) [21] – an otherwise biologically inessential rewarding behavior. This observation is generally consistent with previous research showing that access to alternative non-drug reward or activity can reduce cocaine self-administration in both rats, monkeys and humans [22]–[27]. Preference for sweet water was not attributable to thirst or drinking behavior per se and was observed despite maximal cocaine stimulation and evidence for robust cocaine sensitization [21] – a well-documented behavioral change associated with persistent alterations in brain glutamate and dopamine synapses [28]. Still even more surprisingly, most rats rapidly abstain from cocaine use in favor of the nondrug alternative following an extended period of cocaine self-administration [21]. Previous research showed that following extended access to cocaine self-administration, rats are more likely to escalate their consumption of cocaine [29], to work harder [30] and to take more risk to seek and/or to obtain cocaine [31]. In addition, the ability of cocaine to reinstate cocaine seeking after extinction – a behavioral phenomenon that has been considerably studied over the past 10 years as a model of relapse or craving [32]-[34] – is also increased following a long period of cocaine self-administration [35]-[38]. Clearly, all these behavioral changes and others [39] betray a consistent increase in the reinforcing and/or incentive value of cocaine following extended drug use; nevertheless, no matter how large is this increase in drug value, it is apparently not sufficient to override preference for the nondrug alternative and promote cocaine preference in rats.

As a whole, these observations show that cocaine use has a surprisingly low relative value in the large majority of rats. The goal of the present series of experiments was to test the reliability and generality of this conclusion and to more precisely define the position of cocaine on the value ladder of rats (i.e., preference rank-ordering of different rewards) [40], [41]. We first sought to compare the results from the choice procedure with those of a different reward assessment method – the progressive ratio (PR) schedule [42]. The PR schedule is the most frequently used method to measure the reward value of both drug and nondrug rewards in experimental animals [43], [44]. In the PR schedule, the maximum amount of work that rats accept to do to get access to a given reward (i.e., the breakpoint), serves as an index of its value. Intuitively, one would expect that rats will work more to get access to their preferred reward (i.e., sweet water). Then, using the choice procedure, we attempted to precisely quantify the size of the difference in reward value between cocaine and sweet water. To achieve this end, we measured the point of indifference (or subjective equality) between the 2 rewards by adjusting the cost and concentration of sweet water [45], [46]. We also estimated the conditioned incentive value of each type of reward by testing rats during extinction [47]. Finally, we performed a retrospective analysis of all choice experiments conducted in the laboratory over the past 5 years to assess the influence of the severity of past cocaine use on preference. Overall, we found that no matter how heavy was past cocaine self-administration, most rats value cocaine poorly and readily abstain from cocaine use when offered the opportunity of making a different choice. Only a minority of rats, fewer than 15% at the highest degree of severity of past cocaine use, prefers cocaine over the alternative nondrug reward, even when hungry and offered a natural sugar (i.e., sucrose) that could relieve their need of calories. The persistence of cocaine preference in the face of high stakes strongly suggests a state of addiction.

## Results

**Figure 1:**
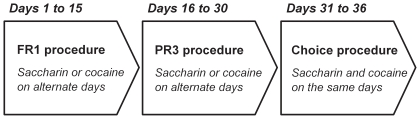
Diagram of the design of the first experiment. For additional information, see the text.

**Figure 2:**
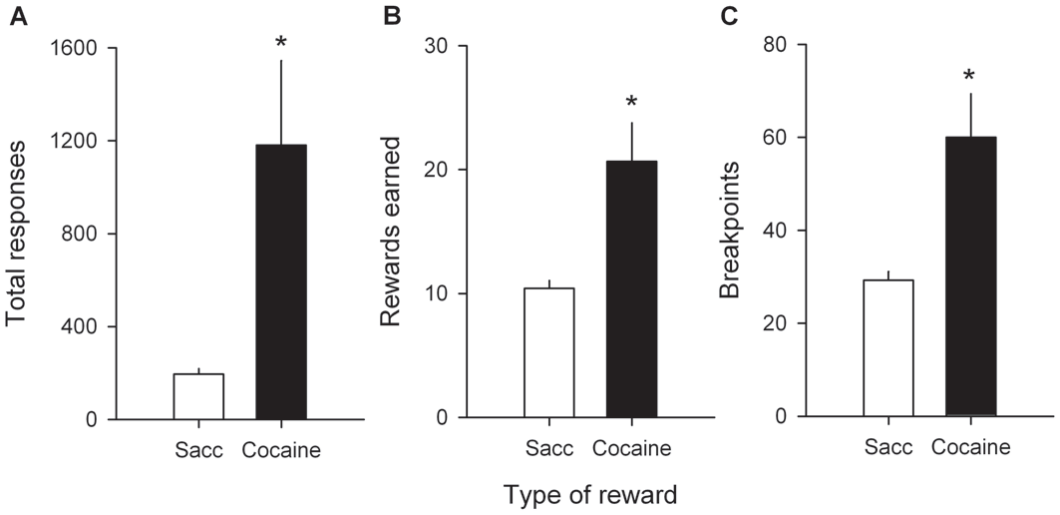
Differential PR responding for cocaine and saccharin. Bars represent the means (± s.e.m.) over the last 3 stable testing sessions of: (A) total responses, (B) rewards earned and (C) breakpoints as a function of reward type [cocaine versus saccharin (sacc)]. *, different from sweet water [*P*<0.01, one-way analysis of variance (ANOVA)].

**Figure 3:**
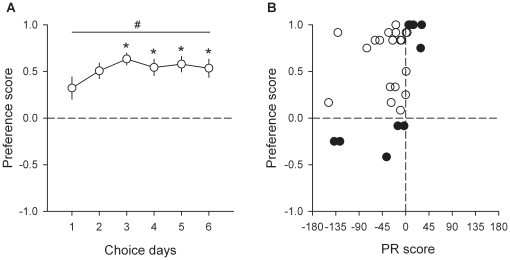
Comparison between reward assessment procedures. (A) Choice between water sweetened with saccharin and cocaine. The horizontal dashed line at 0 indicates the indifference level. Values above 0 indicate a preference for sweet water while values below 0 indicate a preference for intravenous cocaine. *, different from the first day (*P*<0.05, Fisher's LSD test following a one-way ANOVA); #, different from the indifference level (*P*<0.05, *t*-test). (B) Correlation between individual PR and preference scores. The x-axis corresponds to the PR score (difference in breakpoints between saccharin and cocaine; see Results) while the y-axis corresponds to the preference score as measured in the choice procedure (see Materials and Methods). The vertical dashed line at 0 indicates that the breakpoint of cocaine was equal to that of sweet water. Values on the left or on the right of this vertical line indicate that the breakpoint of cocaine is higher or lower than the breakpoint of sweet water, respectively. Open circles represent individuals whose PR and preference scores are incongruent; closed circles represent individuals whose PR and preference scores are congruent. Note that rats with a PR score ≥-3 or ≤3 (i.e., only one step size in the PR3 schedule) were considered to work equally for both types of reward.

Twenty-nine rats from 2 independent cohorts were first trained on alternate daily sessions to lever press to self-administer either water sweetened with saccharin (0.2%) or intravenous cocaine (0.25 mg) under a fixed-ratio 1 (FR) schedule (i.e., one response results in one reward) (see Figure 1 and Materials and Methods). After acquisition and stabilization of FR performance, they were tested alternatively under a progressive-ratio 3 (PR) schedule (i.e., response requirement is increased within-session in constant step of 3 after each successive reward) of either sweet water or cocaine self-administration to measure the breakpoint of each type of reward (see Figure 1 and Materials and Methods). Finally, after stabilization of PR performance, the same rats were tested in the discrete-trials choice procedure to assess individual preferences (see Figure 1 and Materials and Methods). In the FR schedule, most rats self-administered the maximum available number of rewards which was limited to 30 per 3-h session. In the PR schedule, rats responded more vigorously for cocaine than for sweet water [*F*(1, 28) = 7.62, *P*<0.01; Figure 2A]. As a result, they earned more cocaine doses than sweet rewards [*F*(1, 28) = 11.38, *P*<0.01; Figure 2B] and the breakpoint of cocaine was two times higher than the breakpoint of sweet water [*F*(1, 28) = 11.4, *P*<0.01; Figure 2C]. At first glance, these findings suggest that cocaine has a higher value compared to the alternative nondrug reward. However, when allowed to choose mutually-exclusively between the two rewards, the same rats that worked harder for cocaine than for sweet water in the PR schedule clearly preferred the latter over the former [from day 1 to 6: *t*(28)>2.69, *P*<0.01; Figure 3A]. The preference for sweet water was evident on the first day of choice and increased thereafter [*F*(5, 140) = 2.54, *P*<0.05].

To further explore the origin of this apparent contradiction between reward assessment procedures, we computed for each individual the difference in breakpoints between water sweetened with saccharin and cocaine, called thereafter the PR score. Positive PR scores indicate that rats worked more for sweet water than for cocaine and negative PR scores indicate the opposite. We then plotted individual PR scores with individual preference scores, as measured under the discrete-trials choice procedure (see Data Analysis in Materials and Methods), and obtained a graph with 2 indifference lines centered at 0, thereby defining 4 quadrants (Figure 3B). Scores below the horizontal indifference line indicates individual rats that prefer cocaine over sweet water (i.e., 5 out of a total of 29; 17.2%); scores on the left of the vertical line indicates rats that work more for cocaine than for sweet water (i.e., 65.5%). Clearly, the majority of individuals (65.5%; open circles) were behaviorally incongruent across reward assessment procedures: they worked more (or about equally) for cocaine than for sweet water in the PR schedule but preferred the latter over the former during choice. Only a minority of individuals (34.5%; closed circles) were behaviorally congruent. This qualitative analysis was confirmed by a linear regression analysis showing that PR scores were a very poor, though significant, predictor of preference scores [*R* ^2^ = 0.15, *F*(1, 27) = 4.82, *P*<0.05].

**Figure 4:**
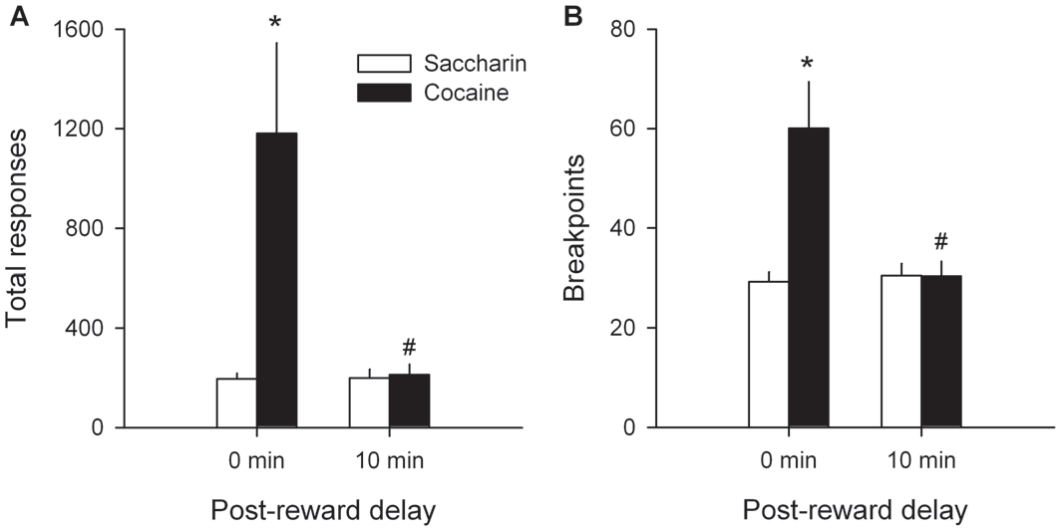
Effects of post-reward delay on PR responding for cocaine. Bars represent the means (± s.e.m.) over the last 3 stable testing sessions of: (A) total responses and (B) breakpoints as a function of reward type (cocaine versus saccharin) and of post-reward delay (0 versus 10 min). *, different from saccharin (*P*<0.01, Fisher's LSD test following a two-way ANOVA); #, different from 0-min delay (*P*<0.01, Fisher's LSD test following a two-way ANOVA).

The contradiction in outcomes between the PR schedule and the choice procedure suggests that these two reward assessment procedures do not entirely measure the same thing. Previous research suggests that responding for cocaine under the PR schedule would not only reflect the value of cocaine but also the direct stimulant effect of cocaine accumulation on work output or effort production [48]–[50]. This latter, value-independent effect should lead to a systematic overestimation of the true value of cocaine in the PR schedule. Note that cocaine accumulation is prevented in the choice procedure by spacing trials with 10-min intervals (see Materials and Methods). Ten minutes is the time that it takes for the dissipation of the stimulant effect of the scheduled dose of cocaine [21]. To test this hypothesis, 23 additional rats from 2 separate cohorts were trained identically as described in the previous experiment, except that the PR schedule was modified as follows: a fixed delay of 10 min was added following each successive reward. During each post-reward delay, the available lever was retracted to avoid extinction. Adding a post-reward delay profoundly decreased responding for cocaine, but not for water sweetened with saccharin, compared to the previous experiment with no delay [Delay X Type of Reward: *F*(1, 50) = 5.84, *P*<0.05; Figure 4A]. As a result, the breakpoint of cocaine decreased to a level comparable to the breakpoint of sweet water which remained constant [Delay X Type of Reward: *F*(1, 50) = 8.85, *P*<0.01; Figure 4B]. This outcome now suggests that the two rewards would be of equal value. However, once again, when the same rats were allowed to choose either cocaine or sweet water, they expressed an immediate and strong preference for sweet water [from day 1 to 6, preference scores were significantly above the indifference line; *t*(22) >4.42, *P*<0.01]. Overall, the first two experiments unexpectedly reveal that the choice procedure is more sensitive and reliable for assessing the relative value of cocaine than the PR schedule, the latter being selectively biased in favor of cocaine.

**Figure 5:**
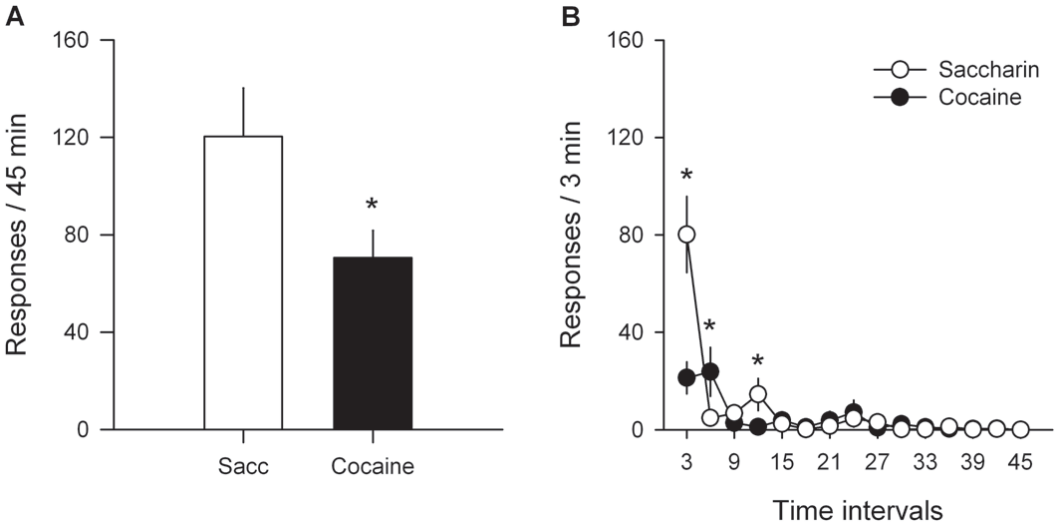
Concurrent extinction of responding for cocaine and saccharin. During extinction testing, the lever previously associated with cocaine was presented concurrently with the lever previously associated with saccharin during 45 min. Pressing on either lever was recorded but had no programmed consequence (no response-contingent reward delivery or light cue presentation). (A) Bars represent the mean total number of responses (± s.e.m.) on the cocaine- and saccharin-associated levers over the 45-min extinction period. *, different from the other reward (*P*<0.05, one-way ANOVA); (B) Curves represent within-session time course of extinction responding on the two levers (means ± s.e.m.). *, different from the other reward (*P*<0.01, Fisher's LSD test following a two-way ANOVA).

To definitively rule out the confounding effect of cocaine accumulation on the assessment of its relative value, difference in responding for cocaine and water sweetened with saccharin was measured during extinction in a separate group of rats (*n* = 12). These rats have previously received over a period of 6 months 59 alternating daily FR sessions of cocaine and saccharin self-administration, followed by 40 alternating daily PR sessions of cocaine and saccharin self-administration which were finally followed by 52 choice sessions. As a result, they had self-administered 1296.7±54.4 intravenous doses of cocaine corresponding to 324.2±13.6 mg of cocaine (which roughly corresponds to 926 mg/kg). During extinction testing, rats had concurrent access for 45 min to the lever associated with cocaine and to the lever associated with water sweetened with saccharin but responding on either lever had no programmed consequence. Thus, during extinction, responding is motivated by the conditioned incentive value that each lever has previously acquired from its associated reward. Consistent with their pre-extinction preference scores [10.4±5.2% cocaine choice, *t*(11) = −7.60, *P*<0.01], but not their pre-extinction PR scores [breakpoint of cocaine: 65.0±7.8; breakpoint of sweet water: 31.6±2.5; *F*(1, 11) = 22.48, *P*<0.01], rats responded more eagerly on the lever associated with sweet water than on the cocaine lever [*F*(1, 11) = 6.88, *P*<0.05; Figure 5A), especially within the first 3 min where the difference in responding on the two levers was the highest [Time X Type of Reward: *F*(14, 154) = 6.74, *P*<0.01; Figure 5B]. This outcome demonstrates that when the direct stimulant effect of cocaine is ruled out, rats work more to attempt to obtain sweet water than cocaine.

**Figure 6:**
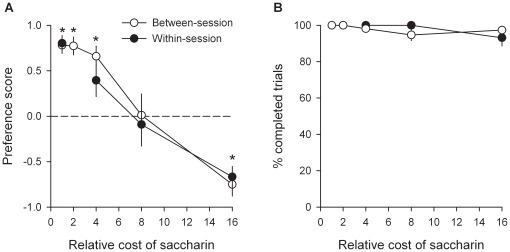
Estimation of the relative value of cocaine. Curves represent (A) choice between cocaine and water sweetened with saccharin and (B) percent of completed trials as a function of the relative cost of saccharin. The cost of saccharin was gradually increased either between sessions (open circles) or within sessions (closed circles). In the former case, each cost level was tested at least 5 times consecutively until stabilization of behavior. Data points represent the means (± s.e.m.) of the last 3 stable testing sessions. For other details, see Materials and Methods, and legend of Figure 3. *, different from the indifference level (*P*<0.05, *t*-test).

**Figure 7:**
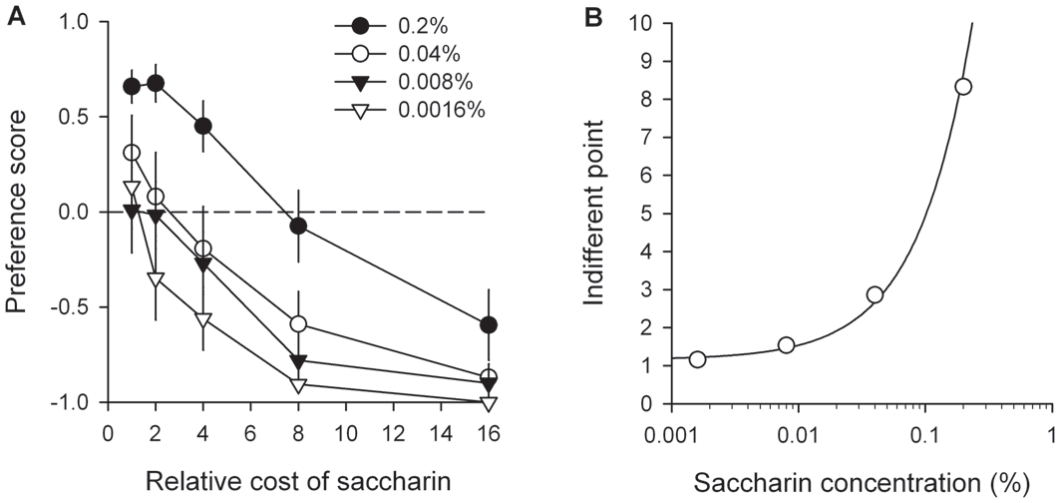
Estimation of the relative value of cocaine as a function of saccharin concentration. Cost-effect curves for each saccharin concentration (A) were established in a within-session manner. Each concentration was tested at least 5 times consecutively until stabilization of behavior. Data curves represent the means (± s.e.m.) of the last 3 stable testing sessions. Indifferent points for each concentration of saccharin (B) were estimated by fitting the corresponding cost-effect curves using a normal sigmoid function. For other details, see Materials and Methods, and legend of Figure 6.

Together with previous research [21], the above series of experiments strongly suggest that for most rats, the reward value of intravenous cocaine is weaker than the value of water sweetened with saccharin. The following series of experiments was aimed at precisely quantifying the magnitude of this difference in reward value using a cost-effect analysis adapted to the choice procedure (see Materials and Methods). In these experiments, rats were first trained to self-administer cocaine or saccharin on alternate days under a FR1 schedule of reinforcement as described above. Then they were tested in the discrete-trials choice procedure during at least 6 consecutive days until stabilization of sweet preference (no increasing or decreasing trend across 3 consecutive days). In the first experiment, which involved 11 rats, after stabilization of preference, the number of responses required to obtain sweet water (or cost) was gradually increased from 1 to 16 times that for cocaine (fixed at 2 responses per reward) until reversal of preference and thus identification of the indifference point. The point of indifference (or also sometimes called the point of subjective equality) corresponds to the relative cost at which rats choose either reward equally (see Materials and Methods). Indifference points provide a continuous common metric to measure and compare the values of rewards as different in kind as intravenous cocaine to sweet water. For instance, if the point of indifference between cocaine and saccharin is equal to X, then one can deduce that the value of cocaine is equal to the value of sweet water when the cost of the latter is X times greater than that of cocaine. As expected, when the cost of water sweetened with saccharin increased, rats progressively shifted their preference to cocaine [*F*(4, 44) = 30.53, *P*<0.01; Figure 6A]. At the highest cost (i.e., 16 times that for cocaine), virtually all rats shifted their preference to cocaine (i.e., 10 out of a total of 11 non drug-preferring rats). Note that the number of completed choice trials was not affected by the cost of saccharin [*F*(4, 44) = 1.6, *NS*; Figure 6B]; this shows that the shift in preference was not influenced by a generalized decrement in performance. Similar results were obtained when the relative cost of sweet water was increased in a within-session manner [*F*(3, 33) = 22.54, *P*<0.01; Figure 6A,B], suggesting that rats made their effort-based decision on a rapid, trial-by-trial reevaluation of the available options. Importantly, in both between- and within-session determinations, the point of indifference was reached when the effort demanded for sweet water was 7.8 (within-session determination, *R^2^* = 0.98, *P*<0.01) to 8.5 (between-session determination, *R^2^* = 0.99, *P*<0.01) times that for cocaine, as estimated by curve fitting of percentage data with a normal sigmoid function (see Materials and Methods). This large relative cost suggests that the value of cocaine is much lower than the value of water sweetened with saccharin. Finally, to further quantify the relative value of cocaine, the point of indifference (or subjective equality) between cocaine and saccharin was measured within-session as a function of the concentration of saccharin (0.0016–0.2%) in an additional group (*n* = 10) of rats. As expected, the cost-effect curve for saccharin preference was shifted to the right with increasing concentrations of saccharin [Saccharin concentration: *F*(3, 27) = 14.26, *P*<0.01; Figure 7A]. As a result, the point of indifference (all *R^2^* were greater than 0.96, *P*<0.01) between cocaine and saccharin increased linearly up to 8.3 with the concentration of saccharin [*R^2^* = 0.988, *P*<0.01; Figure 7B]. Of particular interest, the point of indifference was near 1 at the lowest saccharin concentration (i.e., 0.0016%), suggesting that on average the value of intravenous cocaine was equal to the value of this low concentration in the majority rats.

**Figure 8:**
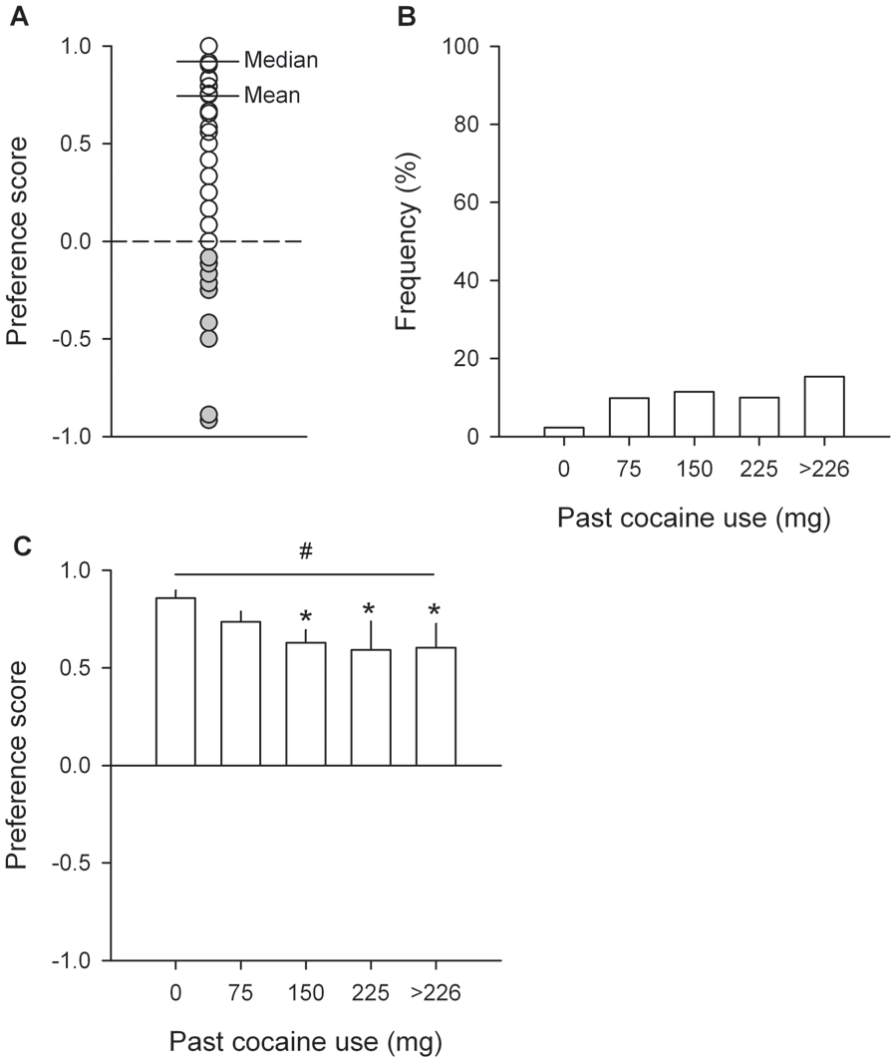
Effects of severity of past cocaine use on cocaine choice. (A) Distribution of individual preferences regardless of past cocaine use. Only 16 individuals out of a total of 184 rats tested in the choice procedure preferred cocaine over water sweetened with saccharin (closed circles). (B) Histograms represent the frequency of cocaine-preferring individuals (i.e., cocaine choices >50% of completed trials over the last 3 stable testing sessions) as a function of past cocaine use (i.e., amount of self-administered cocaine prior to choice testing). (C) Bars represent mean (± s.e.m.) preference over the last 3 stable testing sessions as a function of past cocaine use. For other details, see Materials and Methods, and legend of Figure 3. #, different from the indifference level (*P*<0.05, *t*-test); *, different from the lowest level of severity (*P*<0.01, Fisher's LSD test following a one-way ANOVA).

Though the large majority of rats prefer sweet water over intravenous cocaine, we consistently detected across experiments the existence of a small minority of cocaine-preferring rats (i.e., cocaine choices >50% of completed trials). To estimate the frequency of cocaine-preferring rats, we conducted a retrospective analysis of all choice experiments conducted in the laboratory over the past 5 years, including most of the rats of the present series of experiments. This analysis reveals that only 16 rats out of a total of 184 (i.e., 8.7%) prefer intravenous cocaine over water sweetened with saccharin. To assess the impact of past cocaine use on the frequency of cocaine-preferring rats, the total amount of self-administered cocaine before choice testing was calculated for each individual. This amount ranged from 0 to 486.8 mg (or approximately 1388 mg/kg) and was divided in 5 equal intervals (i.e., of 75 mg each, except for the last open interval), thereby defining 5 increasing levels of severity of past cocaine use (Figure 8A). The frequency of cocaine-preferring individuals increased slightly but not significantly with severity of past cocaine use [Kruskal-Wallis, *H*(4, 184) = 3.47)] and remained below 15% (Figure 8B). Similarly, though the preference for sweet water slightly decreased with the severity of past cocaine use, there was clearly no shift in preference, even at the highest degree of severity [*F*(4, 179) = 2.42, *P*<0.05; Figure 8C]. Thus, no matter how heavy is past cocaine self-administration, cocaine preference in rats remains rare and exceptional.

**Figure 9:**
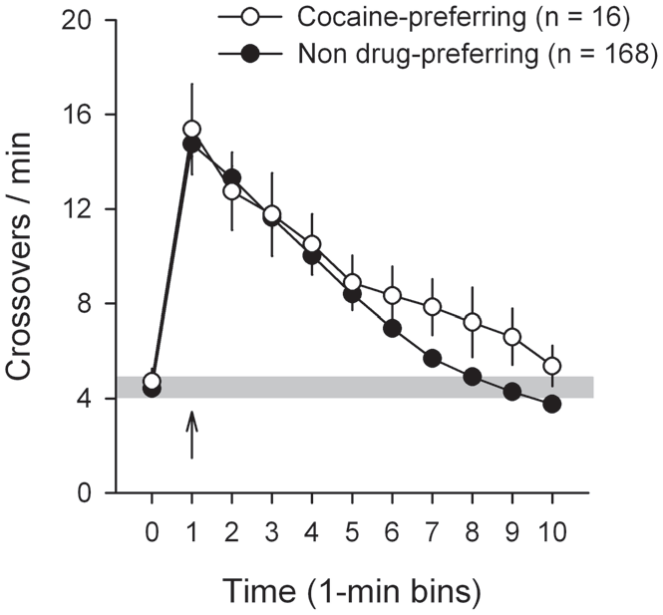
Cocaine-induced locomotion as a function of individual preference. Locomotion (i.e., mean number of cage crossings per min ± s.e.m.) was measured during 10 min after the first cocaine sampling (0.25 mg, i.v.) and was averaged across the last 3 stable choice sessions for each individual. The arrow indicates the intravenous injection of cocaine. The shaded area indicates the mean pre-injection level of locomotion (± s.e.m.). Note that the first cocaine sampling was followed 10 min later by the first saccharin sampling.

**Figure 10:**
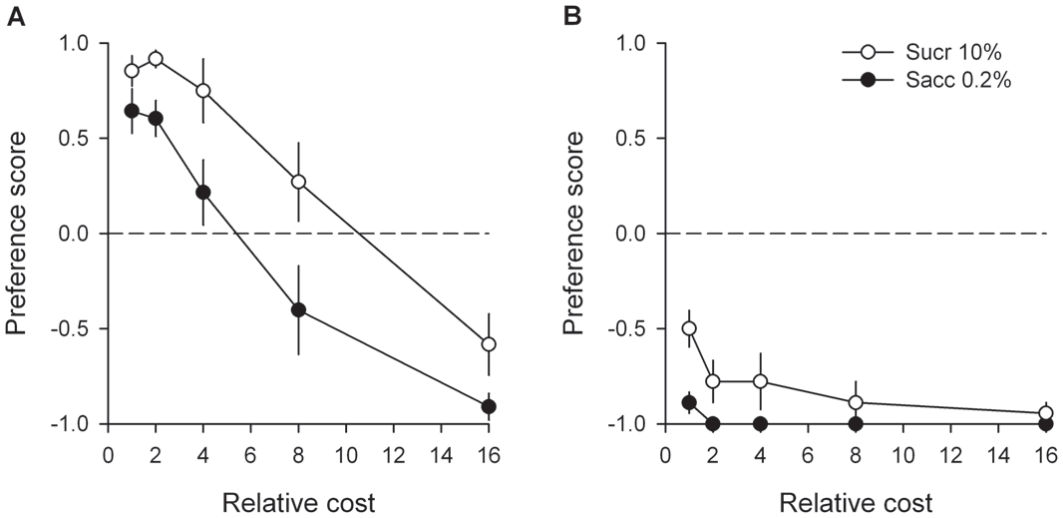
Effects of food restriction on cocaine preference. Cost-effect curves for saccharin (0.2%) or sucrose (10%) were established in a within-session manner in both (A) hungry non drug-preferring (*n* = 8) and (B) hungry cocaine-preferring rats (*n* = 3). Each sweetener was tested at least 5 times consecutively until stabilization of behavior. Data curves represent the means (± s.e.m.) of the last 3 stable testing sessions. For other details, see Materials and Methods, and legend of Figure 6.

Importantly, cocaine preference in cocaine-preferring rats was not attributable to a mere lack of interest in or aversion to water sweetened with saccharin since during saccharin sampling trials, these rats drank as much as the majority of other rats (0.28±0.02 versus 0.31±0.01 ml per 20-s access). In contrast, during cocaine sampling trials, cocaine-preferring rats responded much faster than the majority of other rats to self-administer cocaine [16.0±7.6 versus 54.1±6.5 s; *F*(4, 179) = 2.42, *P*<0.05], suggesting a greater avidity for the drug. This relative avidity for cocaine in cocaine-preferring rats was not due to an increased sensitivity to the psychomotor effects of intravenous cocaine [Group: *F*(1, 182) = 1.09, Group x Time: *F*(9, 1638) = 1.72; Figure 9], as measured following the first cocaine sampling averaged over the last 3 stable testing sessions. Finally, to better determine the strength of cocaine preference, a subgroup of cocaine-preferring rats (*n* = 3) with a history of FR1 training (24 alternating daily sessions of cocaine and saccharin self-administration) and choice testing (36 daily sessions) was chronically food-restricted (i.e., 85% of their free-food body weight) and allowed to choose between cocaine and saccharin (0.2%) and then between cocaine and sucrose (10%) – a natural caloric sugar. The goal of substituting saccharin by sucrose in food-restricted rats was to increase the value and stake of sweet water by increasing its physiological utility (i.e., relief of caloric need). Consistent with previous research [51], we showed in a pilot study that food-restricted rats largely prefer and work harder to obtain sucrose (5–20%) than the highest concentration of saccharin tested (0.2%) (Eric Augier and Serge Ahmed, unpublished data). In addition, in a parallel subgroup of food-restricted, non-drug preferring rats (*n* = 8, same cohort and behavioral history as the 3 cocaine-preferring rats described above), sucrose shifted both downward and rightward the cost-effect curve for sweet preference over cocaine [Type of sweetener: *F*(1, 7) = 21.62, *P*<0.01; Figure 10A]. As a result, the point of indifference between the two rewards increased from about 5.5 to 10.6, suggesting that sucrose plus the need for calories almost doubled the value of sweet water compared to cocaine. In contrast, in cocaine-preferring rats, sucrose did not change significantly the preference for cocaine despite the need for calories [Type of sweetener: *F*(1, 2) = 15.43; Figure 10B].

## Discussion

Several important features of the present series of experiments need to be explicitly stated at the outset to avoid subsequent confusion and/or misinterpretation. First, except for the last experiment with sucrose, rats were neither food or water-deprived throughout experimental testing, so the preference for sweet water – the alternative nondrug reward – over cocaine reported here is not attributable to hunger or thirst. Second, in the present study, rats were first trained to self-administer cocaine and sweet water on several alternate days before being tested in the choice procedure. This initial training clearly showed that rats readily self-administer intravenous cocaine when no other choice is available – as amply demonstrated in previous research [29], [31], [47], [52]. Third, in the discrete-trials choice procedure, rats were allowed to choose either cocaine or water sweetened with saccharin (i.e., choice was mutually-exclusive or either/or). As a result, selecting one reward excluded the alternative reward, thereby allowing individual rats to express their preference. In other words, selecting one reward was equivalent to a renunciation of the alternative reward. In terms of opportunity costs, the cost of selecting one reward corresponded to the loss of opportunity of obtaining the other reward. Fourth, the number of choice trials was restricted to only 8 per day to prevent the eventual confounding effect of differential reward satiation on assessment of reward value [53]. However, in a pilot study, we found that increasing the number of daily choice trials up to 40 had no significant impact on sweet preference (Sarah Dubreucq, Lauriane Cantin and Serge Ahmed, unpublished results). Fifth, trials were spaced by at least 10 min to reduce the direct anorexigenic effect of cocaine accumulation on ingestive behavior – an effect that would obviously bias choice in favor of cocaine, as suggested in other research [54]. However, as shown here, this precaution was superfluous because most rats spontaneously choose not to continue taking cocaine. Note that trial spacing in itself is not the cause of rats' relative lack of interest in cocaine. When no other choice is available, rats self-administer cocaine with forced inter-dose intervals of 10 min or even longer [21], [55]. Finally, the unit dose of cocaine tested in the series of experiments described above (i.e., 0.25 mg per infusion) is a moderate to high dose that has been extensively used in previous research in rats [29], [38], [56]. In fact, as shown in a previous study, most rats continued to prefer water sweetened with saccharin even when the unit dose of cocaine was increased 6-fold, from 0.25 up to the sub-convulsive dose of 1.5 mg [21]. Importantly, the lack of effects of cocaine doses on sweet preference was also seen following extended drug use and escalation of intake, suggesting that the maximal value of cocaine is lower than the value of sweet water [21]. These findings explain why the remainder of this discussion is focused on the relative value of cocaine independently of its dose.

**Figure 11:**
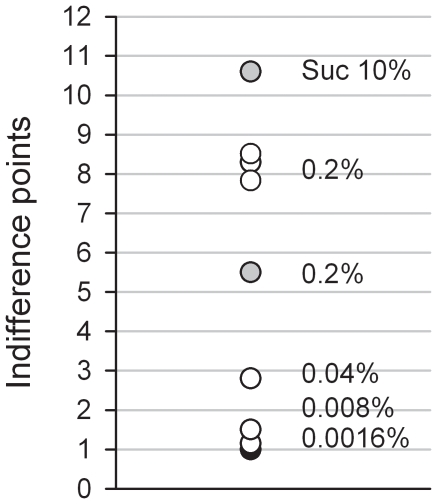
Position of cocaine on the value ladder of rats. Indifference points between cocaine and other types of reward (i.e., different concentrations of saccharin; sucrose) are measured in the same units (i.e., X times the cost of cocaine) and can thus be reported on the same scale. It is reasonably assumed that the indifference point between cocaine and cocaine is 1 (indicated in the graph by the closed circle at the bottom of the scale). Open and gray circles represent indifferent points measured in non-restricted and food-restricted rats, respectively. Note the reproducibility across different experiments (*n* = 3) of the measurements of the indifference point between cocaine and the highest concentration of saccharin (0.2%).

Overall and considering the above information, the present study shows that no matter how heavy was past cocaine self-administration, the large majority of rats readily and almost completely give up cocaine use to engage in another rewarding activity that is biologically inessential (i.e., drinking water sweetened with a non-caloric sweetener is not essential for growth, survival and/or reproduction). Only a small minority of rats, fewer than 15% at the highest degree of severity of past cocaine use, continue to take cocaine despite the opportunity of making a different choice. Importantly, these few rats continued to prefer cocaine, even when hungry and offered a natural sugar (i.e., sucrose) that could relieve their need of calories, a behavior that recalls drug addiction (i.e., continued drug use at the expense of other important activities or occupations). In contrast, the rapid, self-initiated abstinence from cocaine use in the large majority of rats strongly suggests that the value of intravenous cocaine is weaker than previously thought. In support of this interpretation, a systematic cost-effect analysis in these rats revealed that cocaine is low on their value ladder, near the lowest concentration of sweet water. This hedonic position can be visualized in a single graph that represents the distribution of the indifference points corresponding to the different alternatives to cocaine tested in the present series of experiments (Figure 11). The low value of cocaine explains why the conditioned incentive value of the lever associated with cocaine, as measured during extinction, remains relatively low, despite more than 1000 repeated cocaine self-administration from this lever. The weak relative value of intravenous cocaine may also explain why in a previous study, a 6-fold increase in cocaine dose (from 0.25 to a maximum of 1.5 mg) was apparently not sufficient to shift preference to cocaine, even following extended access to cocaine self-administration [21]. Finally, it may also contribute to explain why to study cocaine preference, it is often necessary to increase the cost of the alternative reward [57], [58]. For instance, in several recent studies in monkeys, the cost of cocaine (i.e., FR10) was much lower than the cost of food (i.e., FR100), thereby favoring cocaine preference [57], [58]. As shown here, when the cost of sweet water is much higher than the cost of cocaine, rats too prefer cocaine.

This pattern of results (i.e., cocaine abstinence in most rats, cocaine preference in few rats) could be interpreted as evidence for resilience and vulnerability to cocaine addiction [16]. Specifically, it could suggest that only a minority of rats would be vulnerable to this disorder among a large majority of resilient ones, that is, individuals that cannot constitutively develop addiction even following extensive drug use. In standard experimental settings with no choice than drug use, resilient rats would take cocaine merely by default of other options. Their behavior would be “merely an expectable reaction” to an abnormal situation (i.e., lack of choice or opportunity) and would not necessarily reflect an underlying addiction-related dysfunction [16]. The interpretation in terms of resilience and vulnerability to addiction maps well with what we know about the epidemiology of drug addiction in general and of cocaine addiction in particular. First, among the general population aged 15–54 years, about 12–16% of those who have ever tried cocaine go on to develop cocaine addiction [59], [60]. Second, among recent-onset cocaine users, only a minority (ranging from 4 to 16% depending on the latent class model selected) become addicted to cocaine within 24 months after initiation of cocaine use [61]. Overall, these epidemiological findings show that the large majority of human cocaine users do not eventually become addicted to the drug, a conclusion that is apparently consistent with the pattern of cocaine choice observed here in rats. It is important to note, however, that the interpretation of these findings in terms of resilience to cocaine addiction is delicate and far from clear at present. It is possible that most human cocaine users do not develop addiction, not because they are resilient, as hypothesized here, but merely because they have not used cocaine sufficiently extensively (e.g., due to non-propitious settings). Ideally, to decide between these two possibilities, one must first selectively identify among people who have ever tried cocaine those who used it extensively and then estimate how many of them are resilient to cocaine addiction (i.e., did not develop addiction despite extensive cocaine use).

Perhaps the closest one could get to this epidemiological ideal was in a now old, though still valid, epidemiological survey of heroin users by Lee Robins and co-workers [62], [63]. This survey reported that the large majority of Vietnam veterans (about 90%) who had used heroin on a chronic basis in Vietnam, even to the point of becoming physically dependent, readily and durably stopped heroin use upon return from war [62]. Only a minority of individuals (i.e., about 10%) continued to use heroin after the war. For soldiers during the Vietnam's war, there was little opportunity and heroin use was a cheap, easily available way to make “life in service bearable”, “enjoyable” and also probably to cope with the stress of war [62]. As a result, soldiers were probably using heroin by default of other rewarding or outlet activities, and not because they lost power to control drug use. This interpretation explains why despite chronic and heavy heroin use and evidence of physical dependence, so many veterans (i.e., 90%) stopped heroin use upon return to home. Thus, despite chronic, heavy heroin consumption, most soldiers remained resistant to heroin addiction. As discussed above, there is currently no equivalent evidence for resilience to cocaine addiction after chronic, heavy cocaine use in humans. However, there is some possible evidence for resilience to addiction-like behavior to chronic dopaminergic medication in Parkinson disease [64], [65]. To compensate for the irreversible loss of midbrain dopamine neurons due to neurodegeneration, Parkinsonian patients receive chronic dopamine replacement therapies, including the dopamine precursor levodopa and direct dopamine agonists. In the course of this chronic treatment, some of these patients eventually develop excessive dopaminergic medication use, despite severe motor and non-motor side effects [64]. This syndrome is often called the dopamine dysregulation syndrome and is currently hypothesized to be akin to a state of drug addiction [65]. It is currently estimated that this syndrome appears only in a small minority of patients chronically treated with dopamine replacement therapies (i.e., fewer than 10%), suggesting thus that the remaining majority is likely to be resilient to this syndrome despite years of dopaminergic medication use.

The hypothesis that in rats, like in humans, only a minority of cocaine users would become addicted to cocaine, even after extensive drug use, was previously reached by other researchers using a different approach [66], [67]. Though innovative and interesting, the validity of this approach should nevertheless be considered with caution. It was based on a circular statistical method that limits a priori and arbitrarily to fewer than 33% the maximum possible frequency of rats with an addiction-like behavior. Specifically, an individual was considered to present a specific addiction-like criterion (e.g., an elevated breakpoint for cocaine in the standard PR procedure) if its score for this criterion was above the 66^th^ percentile of the distribution. Obviously, such a frequency-dependent method of identification presupposes at the outset that addiction-like behavior can only affect a minority of rats, with a predefined maximal frequency of 33%. Adding other frequency-dependent criteria could only further decrease this frequency in proportion to the degree of rank correlation between the chosen criteria. Thus, when applied, this method can only identify few rats with addition-like behavior. The fact that it cannot by design allow for a different outcome raises concerns about its validity in objectively measuring the frequency of rats that are resilient or vulnerable to addiction-like behavior. In contrast, the choice-based method of selection advocated here does not set arbitrarily and in advance a limit to the maximum possible frequency of cocaine-preferring rats. In principle, this frequency could attain 100%. The fact that the observed maximum frequency was much lower (i.e., ∼15%) could objectively demonstrate, rather than presuppose, that cocaine addiction only affects a minority of individuals among a sea of resilient ones. Thus, from a methodological standpoint, the choice procedure described here could serve as a reliable sieve for cocaine addiction: it would weed out the majority of resilient rats and only retain the few rats that are potentially addicted to cocaine [16]. In support of the validity of this choice-based method of selection, a recent laboratory study in humans showed that when given a choice between cocaine and money, cocaine users with a DSM-based diagnosis of dependence choose cocaine more frequently than non-addicted long-term cocaine users, regardless of the amount of money available [68].

The present findings have several potential implications for future research in animal models of drug addiction. First, previous research on the neurobiology of drug addiction did not distinguish among animals with extensive cocaine use the minority that is vulnerable to addiction from the majority that is resilient [16]. As a result, brain changes associated with extensive cocaine use are difficult to interpret and their significance for the neurobiology of cocaine addiction is uncertain. In fact, since resilient animals appear to represent a large majority, it is likely that many of these brain changes do not represent neurobiological correlates of addiction but rather other, perhaps normal, neuroplastic adaptations to the novel, salient and unique experience of repeated cocaine use. One way to clarify this important issue in future neurobiological research would be to systematically compare and contrast the minority of vulnerable rats with the resilient majority. Such comparisons could indeed bring unprecedented insights into the neurobiological dysfunctions that are hypothesized to underlie cocaine addiction. Second, another related implication of the present findings is their relevance to preclinical models of cocaine self-administration for the development of medications to treat cocaine addiction. Despite many hopes and promises, experimental research on animal models of drug addiction has had so far only a modest translational impact. This research identified many potential pharmacological targets but no effective treatment for cocaine addiction [69]. Thus, more is clearly needed to improve the predictive validity of preclinical self-administration models in medication development for addiction. In this context, screening medications for their ability to decrease cocaine choice in the small subset of rats that prefer cocaine may better predict their therapeutic efficacy in cocaine-addicted humans.

One of the original goals of the present study was to confirm the weaker value of cocaine, as estimated in the discrete-trials choice procedure, using the classic PR schedule. Paradoxically, we found that though most rats largely prefer sweet water over intravenous cocaine, they nevertheless work harder to obtain the latter than the former. Superficially, this outcome recalls the well-documented “preference reversal” phenomenon in economic decision-making research in humans (i.e., subjects prefer the economic option that they valued less in independent evaluation) [70]. Additional investigation, however, showed that this apparent paradox results from a selective bias in the PR schedule of cocaine self-administration. Contrary to the breakpoint of sweet water which only depends on the value of this reward, the breakpoint of cocaine depends on two independent effects: the reward value of the scheduled dose of cocaine and the direct stimulant effect of cocaine accumulation on work output or effort production [48], [50]. When the latter, value-independent effect of cocaine is minimized by reducing cocaine accumulation with forced spaced trials, the breakpoint of cocaine considerably decreases, a finding that is consistent with previous research in monkeys [49], [71]. Importantly, spacing access to sweet water had no similar impact. Thus, the breakpoint of cocaine, as measured in the standard PR schedule, provides a biased overestimate of the value of cocaine that partly explains the apparent discrepancy with the choice procedure. It is possible that with more spaced PR trials (i.e., greater than 10 min), the breakpoint of cocaine could have decreased below that of sweet water – a prediction that warrants further research. This selective bias probably also explains why the breakpoint of cocaine is generally much higher than that of other, non-stimulant drugs (e.g., heroin; nicotine) which nevertheless are equally or even more addictive than cocaine in humans [72]–[74]. Thus, the present series of experiments unexpectedly reveals that the standard PR schedule is selectively biased in favor of cocaine and is thus less suited than the choice procedure to assess its relative value. Nevertheless, it is worth mentioning here that although the present study demonstrates the importance of cocaine's stimulant properties in the very high cocaine breakpoints typically achieved in the standard PR schedule, humans tend to self-administer cocaine in a similar binge pattern, with relatively short intervals between successive doses. Thus, perhaps it is most valid, for certain research questions, to study a short inter-dose interval of self-administration in rats, even though the resulting breakpoint reflects both reinforcement and stimulant effects.

Finally, despite many advantages, the choice-based method of identification of individuals that are vulnerable or resilient to drug addiction has also some potential limitations. Perhaps the most important limitation is that lack of drug preference alone is not always sufficient evidence for ruling out cocaine addiction. For instance, in the case of polysubstance addiction, preference for one substance does not rule out addiction to the other substance. It merely indicates that one addiction is stronger than the other. In the present study, if rats happened to be addicted to both sweet water and cocaine, then sweet preference would only indicate that addiction to sweet water is stronger than cocaine addiction. However, though there is growing evidence for food and sugar addiction in both animals and humans [75]–[78], co-addiction to sweet water and cocaine is unlikely to explain the pattern of cocaine choice reported here. In a previous study, rats with extensive cocaine use shifted their preference to sweet water within only two days and after having drunk less than 5 ml of sweet water [21]. It seems very unlikely that most rats could become addicted to sweet water so rapidly and following such a low level of consumption. In addition, recent estimation in humans suggests that food addiction, like cocaine addiction, would only affect a minority of people [76]. Finally and more generally, one must consider in interpreting the present findings that preference alone is also probably not sufficient evidence for inferring a state of addiction. What also counts is the opportunity costs or negative consequences associated with a preference. For instance, if one demonstrated that female rats systematically prefer their pups over cocaine, one would rightly not consider this preference as reflecting addiction. Maternal preference for pups is a normal, expectable behavior in female rats and the associated renunciation of cocaine use is not a major cost. In contrast, however, if few female rats preferred cocaine to the detriment of the welfare and/or survival of their pups, then one would be founded in interpreting such preference as possible evidence for addiction-like behavior [79]–[81]. Indeed, in this case, the opportunity cost is relatively severe as it leads to a reduction in biological fitness. In the present study, preference for cocaine was associated with reduced welfare, as it persisted even when rats were hungry and offered a natural sugar (i.e., sucrose) that could relieve their need of calories. The persistence of cocaine preference in the face of high stakes strongly suggests a state of addiction.

## Materials and Methods

### Ethics statement

All experiments were carried out in accordance with institutional and international standards of care and use of laboratory animals [UK Animals (Scientific Procedures) Act, 1986; and associated guidelines; the European Communities Council Directive (86/609/EEC, 24 November 1986) and the French Directives concerning the use of laboratory animals (décret 87–848, 19 October 1987)]. All experiments have been approved by the Committee of the Veterinary Services Gironde, agreement number B-33-063-5, 13 June 2006.

### Subjects

Naïve, young adult (2 months and a half old at the beginning of experiments), male, Wistar rats (*n* = 83, Charles River, France) completed the present study. Rats were housed in groups of two or three and were maintained in a light- (12-h reverse light-dark cycle) and temperature-controlled vivarium (22°C). All behavioral testing occurred during the dark phase of the light-dark cycle. Food and water were freely available in the home cages, except when specified below. Food consisted of standard rat chow A04 (SAFE, Scientific Animal Food and Engineering, Augy, France) that contained 60% of carbohydrates (largely corn starch), 16% of proteins, 12% of water, 5% of minerals, 3% of fat and 4% of cellulose. No synthetic or refined sugar was added.

### Apparatus

Twelve identical operant chambers (30×40×36 cm) were used for all behavioral training and testing (Imétronic, France). All chambers were located away from the colony room in a dimly lit room. They were individually enclosed in wooden cubicles equipped with a white noise speaker (45±6 dB) for sound-attenuation and an exhaust fan for ventilation. Each chamber had a stainless-steel grid floor that allowed waste collection in a removable tray containing maize sawdust. Each chamber was constituted of two opaque operant panels on the right and left sides, and two clear Plexiglas walls on the rear and front sides (the front side corresponds to the entry/exit of the chamber). Each operant panel contained an automatically-retractable lever, mounted on the midline and 7 cm above the grid. The left operant panel was also equipped with a retractable, cylinder-shaped drinking spout, 9.5 cm to the left of the lever and 6 cm above the grid. A lickometer circuit allowed monitoring and recording of licking. A white light diode (1.2 cm OD) was mounted 8.51cm above each lever (from the center of the diode). Each chamber was also equipped with two syringe pumps placed outside, on the top of the cubicle. One syringe pump was controlled by the left lever and delivered water sweetened with saccharin solution into the drinking spout through a silastic tubing (Dow Corning Corporation, Michigan, USA). The other pump was controlled by the right lever and delivered drug solution through a Tygon tubing (Cole Parmer) connected via a single-channel liquid swivel (Lomir biomedical inc., Quebec, Canada) to a cannula connector (Plastics One, Roanoke, VA) on the back of the animal. The Tygon tubing was protected by a stainless-steel spring (0.3 cm ID, 0.5 cm OD) (Aquitaine Ressort, France) which was suspended at the center of the chamber from the swivel tether connector. Vertical movements of the animal were compensated for by means of a counterbalancing weight-pulley device.

### Surgery

Anesthetized rats [chloral hydrate (500 mg/kg, i.p., J-T Baker, The Netherlands) or a mixture of xylazine (15 mg/kg, i.p., Merial, France) and ketamine (110 mg/kg, i.p., Bayer Pharma, France)] were surgically prepared with silastic catheters (Dow Corning Corporation, Michigan, USA) in the right jugular vein that exited the skin in the middle of the back about 2 cm below the scapulae. After surgery, catheters were flushed daily with 0.15 ml of a sterile antibiotic solution containing heparinized saline (280 IU/ml) (Sanofi-Synthelabo, France) and ampicilline (Panpharma, France). When a catheter leakage was suspected, the patency of the catheter was checked by an intravenous administration of etomidate (1 mg/kg, Braun Medical, France), a short-acting non-barbiturate anesthetic. Behavioral testing began 7–10 days after surgery.

### Fixed-ratio schedule

Operant- and drug naïve rats were trained under a fixed-ratio 1 (FR1) schedule of saccharin and cocaine self-administration on alternate daily sessions, six days a week. On saccharin sessions, the lever associated with saccharin was extended to mark the onset of the session and to signal saccharin availability; the other lever remained retracted. One lever pressing on the extended lever was rewarded by a 20-s access to water sweetened with 0.2% of sodium saccharin delivered in the adjacent drinking cup and initiated a concomitant 20-s time-out period signaled by the illumination of the cue-light above the lever. During the time-out period, responding had no scheduled consequences. The first 3 s of each 20-s access to sweet water, the drinking cup was filled automatically with sweet water; during the next 17 s, additional volumes of sweet water were obtained on demand by voluntary licking (approximately 0.02 ml per 10 licks). Note that 20 s of access to sweet water is a short access. When given free access to sweet water, rats can drink almost continuously during 20–30 minutes before reaching satiety (Magalie Lenoir and Serge Ahmed, unpublished observations). On cocaine sessions, the lever associated with cocaine was extended to mark the onset of the session and to signal cocaine availability; the lever associated with saccharin remained retracted. One lever pressing on the extended lever was rewarded by one intravenous dose of 0.25 mg cocaine in a volume of 0.15 ml delivered over 4 s and initiated a concomitant 20-s time-out period signaled by the illumination of the cue-light above the lever. During the time-out period, responding had no scheduled consequences. The dose of cocaine has been widely used in previous research on cocaine self-administration, including our own research. Sessions ended after rats had earned a maximum of 30 saccharin or cocaine rewards or 3 h had elapsed.

### Progressive-ratio schedule

Following training in the FR schedule, rats were tested under a linear progressive-ratio (PR) schedule of saccharin or cocaine self-administration on alternate daily sessions, six days a week. All experimental conditions were identical to those used in the FR schedule, except that the response requirement or cost was increased within-session by a constant increment of 3 following each sweet or cocaine reward (i.e., 1, 4, 7, 10…). PR sessions terminated after 30 min had elapsed without a reward or 4 h had elapsed. After stabilization of performance, PR sessions ceased within 3 h for most rats (i.e., over 90%). The break point was defined as the last completed response requirement and corresponded to the total number of rewards earned during the PR session.

### Discrete-trials choice procedure

Rats were allowed to choose during several consecutive daily sessions between the lever associated with cocaine (lever C) and the lever associated with water sweetened with saccharin (lever S) on a discrete-trials choice procedure. Each daily choice session consisted of 12 discrete trials, spaced by 10 min, and divided into two successive phases, sampling (4 trials) and choice (8 trials). During sampling, each trial began with the presentation of one single lever in this alternative order: C – S – C – S. Lever C was presented first to prevent an eventual drug-induced taste aversion conditioning or negative affective contrast effects. If rats responded within 5 min on the available lever, they were rewarded by the corresponding reward (i.e., 0.25 mg cocaine delivered intravenously or 20-s access to water sweetened with 0.2% saccharin, as described above). Reward delivery was signaled by retraction of the lever and a 40-s illumination of the cue-light above this lever. If rats failed to respond within 5 min, the lever retracted and no cue-light or reward was delivered. Thus, during sampling, rats were allowed to separately evaluate each reward before making their choice. During choice, each trial began with the simultaneous presentation of both levers S and C. Rats had to select one of the two levers. During choice, reward delivery was signaled by retraction of both levers and a 40-s illumination of the cue-light above the selected lever. If rats failed to respond on either lever within 5 min, both levers retracted and no cue-light or reward was delivered. The response requirement of each reward was set to 2 consecutive responses to avoid eventual accidental choice. A response on the alternate lever before satisfaction of the response requirement reset it. Response resetting occurred very rarely, however.

### Quantitative assessment of the relative value of cocaine: between-session determination

After stabilization of preference (i.e., no increasing or decreasing trends over 3 consecutive days), the number of responses or cost required to obtain water sweetened with saccharin – the preferred reward – was gradually incremented between sessions from 1 to 16 times that for cocaine which remained constant (i.e., 2 responses per reward). The goal was to produce a shift in preference to measure the point of indifference (or subjective equality) between the 2 rewards. Each level of cost was tested for at least 5 consecutive sessions and until stabilization of choice performance. The point of indifference between the 2 rewards was estimated by fitting the (group-average) cost-effect curve with a normal (i.e., three-parameter) sigmoid function (least-squares non-linear regressions, Sigmaplot 2002, version 8.02). For curve fitting, data were expressed in percentage of cocaine choices with the maximum set at 100%. Graphically, the indifference point corresponds thus to the relative cost of the alternative at which the fitted curve crosses the indifference line of 50%.

### Quantitative assessment of the relative value of cocaine: within-session determination

After stabilization of preference, the relative cost of sweet water – the preferred reward – was gradually increased in a within-session manner every 4 choice trials. In the first within-session cost-effect analysis which was conducted in the same rats following the between-session analysis, there were a total of 16 discrete choice trials, corresponding to 4 levels of cost of sweet water: 1, 4, 8 and 16 times the cost of cocaine in this order. In all subsequent within-session cost-effect analyses, each daily session consisted of 4 sampling trials, as in the standard procedure, followed by 20 discrete choice trials, corresponding to 5 levels of relative cost: 1, 2, 4, 8 and 16 times the cost of cocaine in this order. Otherwise experimental conditions were identical to those in the standard choice procedure. For each tested variable (e.g., saccharin concentration), rats were tested for at least 5 consecutive sessions and until stabilization of the within-session cost-effect curve. The point of indifference between cocaine and sweet water was estimated by curve fitting as described above.

### Retrospective analysis of the frequency of cocaine-preferring individuals

Over the past 5 years, a total of 184 rats belonging to 13 independent cohorts were tested in the choice procedure described above during at least 5 consecutive daily sessions until behavioral stabilization (i.e., 3 consecutive sessions with more than 50% of completed choice trials [range: 58 to 100%; median: 100] and without decreasing or increasing trends in preference score; see also, Data Analysis). Data from some of these rats were published elsewhere [21], though not under this form (i.e., frequencies) and not as a function of past cocaine use. These rats had a wide variety of history of cocaine self-administration before choice testing, ranging from no prior exposure to extended exposure to cocaine self-administration. As a result, the amount of self-administered cocaine ranged from 0 to 486 mg (or approximately 1388 mg/kg) and defined 5 levels of severity: 0 (*n* = 43), 1–75 (*n* = 66), 76–150 (*n* = 52), 151–225 (*n* = 10), >226 mg (*n* = 13). Then, we estimated the frequency of cocaine-preferring rats by counting for each degree of severity the number of individuals with a preference score below 0 (i.e., cocaine choices >50% of trials over 3 stable sessions; see Data Analysis).

### Drugs

Cocaine hydrochloride (Coopération Pharmaceutique Française, France) was dissolved in 500-ml sterile bags of 0.9% NaCl and kept at room temperature (21±2°C). Drug doses were expressed as the weight of the salt. Sodium saccharin (Sigma-Aldrich, France) or sucrose (Sigma-Aldrich, France) was dissolved in tap water at room temperature (21±2°C). Sweet solutions were renewed each day.

### Data Analysis

The indifference level between water sweetened with saccharin (or sucrose) and cocaine was conveniently normalized at 0 in the discrete-trials choice procedure. Scores above 0 indicated a preference for the nondrug alternative (i.e., selection of this reward >50% of completed choice trials) while scores below 0 indicated a preference for cocaine (i.e., selection of this reward >50% of completed choice trials). In the PR schedule, scores correspond to the difference in breakpoints between the nondrug alternative and cocaine. Individuals with a PR score between −3 and +3 (i.e., corresponding to a difference of one step size in the PR3 schedule) were considered to work equally for both types of reward. Statistical analyses were run using Statistica, version 7.1 (Statsoft, Inc France).
